# 4146 Establishment of Screening Methods for G6DP Deficiency – Translational and Clinical Applications

**DOI:** 10.1017/cts.2020.332

**Published:** 2020-07-29

**Authors:** Christian Gomez, Ingrid C. Espinoza, Kerri A. Harrison, Fremel J. Backus, Krishna K. Ayyalasomayajula, Kim G. Adcock, Lisa M. Stempak, Richard L. Summers, Leigh Ann Ross, Larry Walker

**Affiliations:** 1 University of Mississippi; 2 University of Mississippi Medical Center; 3 Case Western Reserve University

## Abstract

OBJECTIVES/GOALS: To develop feasible screening methods for activity of the enzyme Glucose-6-phosphate dehydrogenase (G6PD) with point of care applicability. METHODS/STUDY POPULATION: Current knowledge establishes the relevance of G6PD as a critical therapeutic determinant for effective antimalarial therapy due to the occurrence of mutations that lead to post-treatment severe adverse effects. We present our findings on development of cost effective point-of-care screening methodologies to ascertain G6PD deficiency. RESULTS/ANTICIPATED RESULTS: Using Patient Cohort Explorer and data from the Department of Pathology, we established the prevalence of G6PD deficiency at the University of Mississippi Medical Center, Jackson, MS as high as 11.8% (African-American males in all population, n = 2518). Next, for selection of potential target groups, we set up a protocol for recruitment of volunteers based on ethnic background, parental ethnicity, and medical history. G6PD activity was evaluated using point of care methods [Trinity Biotech test or CareSTART Biosensor], and Gold Standard quantitative spectrophotometric assay (LabCorp). Determinations in >20 subjects have showed comparable concordance. If used with a conservative interpretation of the signal, the Trinity Biotech test showed superior potential for use in the field relative to the CareSTART Biosensor. DISCUSSION/SIGNIFICANCE OF IMPACT: We established the prevalence of G6PD deficiency in our medical center. We have also setup tests for point-of-care assessment of G6PD. Pending evaluation of the relative tests performance, we will be in position to screen individuals and select them for a prospective clinical trial to evaluate the safety of antimalarial agents on scope of G6PD deficiency.

